# The Scleraxis Transcription Factor Directly Regulates Multiple Distinct Molecular and Cellular Processes During Early Tendon Cell Differentiation

**DOI:** 10.3389/fcell.2021.654397

**Published:** 2021-06-03

**Authors:** Han Liu, Jingyue Xu, Yu Lan, Hee-Woong Lim, Rulang Jiang

**Affiliations:** ^1^Division of Developmental Biology, Cincinnati Children’s Hospital Medical Center, Cincinnati, OH, United States; ^2^Division of Plastic Surgery, Cincinnati Children’s Hospital Medical Center, Cincinnati, OH, United States; ^3^Department of Surgery, University of Cincinnati College of Medicine, Cincinnati, OH, United States; ^4^Department of Pediatrics, University of Cincinnati College of Medicine, Cincinnati, OH, United States; ^5^Division of Biomedical Informatics, Cincinnati Children’s Hospital Medical Center, Cincinnati, OH, United States

**Keywords:** bHLH, cell differentiation, ChIP-seq, CRISPR, RNA-seq, Scx, tendon development

## Abstract

Proper development of tendons is crucial for the integration and function of the musculoskeletal system. Currently little is known about the molecular mechanisms controlling tendon development and tendon cell differentiation. The transcription factor Scleraxis (Scx) is expressed throughout tendon development and plays essential roles in both embryonic tendon development and adult tendon healing, but few direct target genes of Scx in tendon development have been reported and genome-wide identification of Scx direct target genes *in vivo* has been lacking. In this study, we have generated a *Scx*^*Flag*^ knockin mouse strain, which produces fully functional endogenous Scx proteins containing a 2xFLAG epitope tag at the carboxy terminus. We mapped the genome-wide Scx binding sites in the developing limb tendon tissues, identifying 12,097 high quality Scx regulatory *cis-*elements in-around 7,520 genes. Comparative analysis with previously reported embryonic tendon cell RNA-seq data identified 490 candidate Scx direct target genes in early tendon development. Furthermore, we characterized a new *Scx* gene-knockout mouse line and performed whole transcriptome RNA sequencing analysis of E15.5 forelimb tendon cells from *Scx*^–/–^ embryos and control littermates, identifying 68 genes whose expression in the developing tendon tissues significantly depended on Scx function. Combined analysis of the ChIP-seq and RNA-seq data yielded 32 direct target genes that required Scx for activation and an additional 17 target genes whose expression was suppressed by Scx during early tendon development. We further analyzed and validated Scx-dependent tendon-specific expression patterns of a subset of the target genes, including *Fmod*, *Kera*, *Htra3*, *Ssc5d*, *Tnmd*, and *Zfp185*, by *in situ* hybridization and real-time quantitative polymerase chain reaction assays. These results provide novel insights into the molecular mechanisms mediating Scx function in tendon development and homeostasis. The ChIP-seq and RNA-seq data provide a rich resource for aiding design of further studies of the mechanisms regulating tendon cell differentiation and tendon tissue regeneration. The *Scx*^*Flag*^ mice provide a valuable new tool for unraveling the molecular mechanisms involving Scx in the protein interaction and gene-regulatory networks underlying many developmental and disease processes.

## Introduction

Tendons and ligaments are specialized connective tissues densely packed with collagen fibers, composed primarily of type I collagen fascicles with several other collagens, elastin, and various proteoglycans making up the remainder of the extracellular matrix (ECM) surrounding the resident tenocytes (Birch et al., 2013; Davis et al., 2013). Tendons connect skeletal muscles to bones and transmit mechanical forces generated from muscle contraction whereas the ligaments align bones within joints and reinforce their stability and flexibility. Many musculoskeletal diseases involve injuries and tissue degeneration in tendons and ligaments (Yang et al., 2013; Nourissat et al., 2015). Although it is expected that many genes and molecular pathways are shared during the processes of tendon development and tendon healing/regeneration, the molecular mechanisms controlling tendon development, including specification of tendon progenitor cells from mesenchymal stem cells, migration to local domains and differentiation into mature tenocytes, secretion of ECM molecules and organization of fibrils and fibers into tendon bundles, are not well understood. With the development and wide application of gene knockout technologies, thousands of mutant mouse lines carrying loss-of-function mutations in individual genes have been generated and analyzed in the last 30 years. However, only loss of function of the Scleraxis (Scx) transcription factor and TGFβ signaling led to severe disruption of tendon development whereas mice deficient in several other genes individually or in combination, including genes encoding the Mohawk (Mkx) transcription factor, the early growth response (EGR) 1 and 2 transcription factors, various proteoglycans, and tenomodulin, exhibited mild postnatal tendon defects (Docheva et al., 2005; Murchison et al., 2007; Kilts et al., 2009; Pryce et al., 2009; Ito et al., 2010; Liu et al., 2010; Lejard et al., 2011; Dourte et al., 2013; Guerquin et al., 2013; Dunkman et al., 2014; Delgado Caceres et al., 2018; Shukunami et al., 2018). While these data suggest that the tendon developmental processes are well orchestrated with built-in compensatory regulatory mechanisms (Delgado Caceres et al., 2018), better understanding of the molecular mechanisms controlling tendon development and tendon cell differentiation will be instrumental for the development of effective methods for tendon repair and regeneration.

Scleraxis is a basic helix-loop-helix (bHLH) transcription factor that is expressed in early embryonic tendon progenitor cells and stays strongly expressed throughout tendon cell differentiation into mature tenocytes (Cserjesi et al., 1995; Schweitzer et al., 2001). Mice homozygous for *Scx* null mutation exhibit severe hypoplasia or complete absence of force transmitting tendons, while the muscle anchoring tendons and ligaments are less affected (Murchison et al., 2007; Shukunami et al., 2018). Further studies showed that Scx function is not required for tendon progenitor cell initiation but is crucial for tendon cell differentiation (Murchison et al., 2007). Studies in mouse and chick embryos showed that TGFβ and FGF signaling pathways are essential for induction of tendon progenitor cells by activating expression of *Scx* and other tendon genes (Kuo et al., 2008; Pryce et al., 2009; Hasson, 2011; Havis et al., 2016). Moreover, a recent study demonstrated that Scx function is also required for adult tendons in response to mechanical loading (Gumucio et al., 2020). How Scx regulates tendon cell differentiation is still unclear. Currently very few genes, including *Col1a1* and *Tnmd*, have been identified as potential direct target genes of Scx in tenocytes primarily through *in vitro* functional analysis of putative Scx-binding promoter elements (Shukunami et al., 2006, 2018; Lejard et al., 2007), whereas large scale identification of Scx direct transcriptional target genes during tendon cell differentiation *in vivo* is still lacking.

In this study, we first generated a novel *Scx*^*Flag*^ knockin mouse line, which expresses the endogenous Scx protein with a 2xFLAG epitope tag at the carboxy terminus, through CRISPR/cas9-mediated genome editing. Using the *Scx*^*Flag*^ mice, we mapped the genome-wide Scx binding sites in embryonic tendon progenitors and differentiating tendon cells using chromatin immunoprecipitation followed by high throughput DNA sequencing, which mapped high quality Scx-binding sites in the promoter and/or enhancer regions in 7,520 genes. Furthermore, we characterized a new *Scx* gene-knockout mouse resource that is accessible to the wide biomedical research community and performed whole transcriptome RNA sequencing analysis of early differentiating limb tendons in *Scx*^–/–^ and wildtype littermates. These ChIP-seq and RNA-seq datasets provide a rich resource for further studies of the molecular mechanisms regulating tendon formation and tendon cell differentiation. Combined analyses of the ChIP-seq and RNA-seq data provide novel insights into the molecular mechanisms mediating Scx regulation of tendon cell differentiation.

## Materials and Methods

### Mice

**FIGURE 1:**
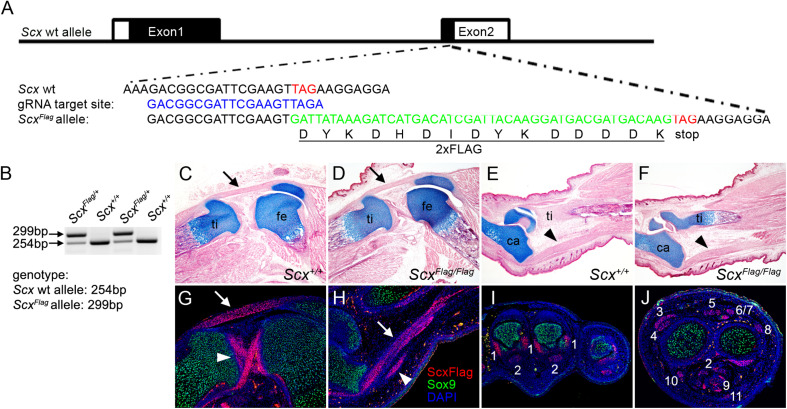
Generation of *Scx*^*Flag*^ mice. **(A)** Schematics of the strategy for generating *Scx*^*Flag*^ mice using the CRISPR/CAS9 approach. The top row shows the genomic organization of the mouse *Scx* locus. The two exons are boxed with coding regions filled in black and untranslated regions in white. The second row shows the sequence around the translation STOP codon (TAG, labeled in red font). The third row shows the selected guide RNA target site sequence. The fourth row shows part of the correctly edited *Scx*^*Flag*^ allele sequence containing the insertion of the 2xFLAG coding sequence (in green font) immediately 5′ to the STOP codon. The fifth row shows the amino acid sequence of the 2xFLAG tag. **(B)** PCR genotyping result from a litter of *Scx*^*Flag*/+^ mouse intercross. **(C–F)** Images of HE and alcian blue-stained longitudinal sections through the middle of the knee **(C,D)** and ankle **(E,F)** of E18.5 wildtype **(C,E)** and *Scx*^*Flag/Flag*^ **(D,F)** embryos. Arrow in panels **(C,D)** point to patellar tendon. Arrowheads in panels **(E,F)** point to Achilles tendon. ca, calcaneus; fe, femur; ti, tibia. **(G–J)** immunofluorescent staining of sections through the knee **(G)**, ankle **(H)**, forelimb autopod **(I)**, and forelimb zeugopod **(J)** regions of E16.5 *Scx*^*Flag*/+^ embryos. Immunofluorescence of Sox9 is shown in green color, Immunofluorescence of the FLAG epitope is shown in red color, and DAPI counterstaining of cellular nuclei is shown in blue color. Arrow in panels **G,H** points to patellar and Achilles tendons, respectively. Arrowhead in panel **(G)** point to the cruciate ligaments, whereas arrowhead in panel **(H)** point to a smaller tendon next to the Achilles. The numbers in panels **(I,J)** mark distinct forelimb tendons as: 1 - Collateral Ligament Metacarpophalangeal joint; 2 - Flexor Digitorium Profundus; 3 - Extensor Pollicis; 4 - Extensor Carpi Radialis Longus; 5 - Extensor Digitorium Communis; 6 - Extensor Digiti Quarti; 7 - Extensor Digiti Quinti; 8 - Extensor Carpi Ulnaris; 9 - Flexor Digitorium Sublimis; 10 - Flexor Carpi Radialis; 11 - Palmaris Longus.

*Scx-GFP* transgenic mice (Pryce et al., 2007) were generously provided by Dr. Ronen Schweitzer (Shriners Hospitals for Children, Portland, Oregon). To generate *Scx*^*Flag*^ knock-in mice, pretested guide RNAs targeting the genomic sequence containing the endogenous Scx translational STOP codon were co-injected with a single-stranded oligonucleotide donor template (Figure 1A) and humanized *Cas9* mRNAs into zygotes of B6D2F1 (C57BL/6 X DBA2) mice. Genome-modified founder mice were identified by using polymerase chain reaction (PCR) assay and crossed to CD-1 mice to generate *Scx*^*Flag*/+^ hemizygous mice. Genotypically verified G1 *Scx*^*Flag*/+^ hemizygous mice were intercrossed to generate *Scx*^*Flag/Flag*^ homozygous mice. *Scx^+/–^* mutant mice were purchased from the Mutant Mouse Research and Resource Center at the University of California Davis (KOMP catalog#13478). All mouse strains were maintained by crossing to CD-1 wildtype mice (Charles River, Stock# 022), or by intercrossing between siblings. All animal procedures were approved by the Institutional Animal Care and Use Committee (IACUC) at Cincinnati Children’s Hospital Medical Center (CCHMC). The mice were housed in AAALAC accredited barrier housing conditions at CCHMC.

### Histology, *in situ* Hybridization, and Immunofluorescent Staining

Mice were euthanized at predetermined stage and embryos were collected in cold PBS, fixed in 4% PFA overnight, washed in PBS for 3 times and processed to 100% methanol (for whole mount *in situ* hybridization), or paraffin for section (for histology staining and section *in situ* hybridization). Histology staining and *in situ* hybridization procedures were performed as previously described (Liu et al., 2013, 2015). *In situ* probe templates were amplified by PCR from total cDNA sample of E13.5 wildtype forelimbs. Antisense RNA probes were synthesized from templates using T7 RNA polymerase (Promega catalog# P2075). Immunofluorescent staining was performed as previously described (Xu et al., 2014). The primary antibodies used include anti-Sox9 (Santa Cruz, catalog# sc-20095, 1:25 dilution) and anti-FLAG antibody (Sigma, catalog# F1804, 1:50 dilution). The secondary antibodies were Alexa Fluor 568-conjugated goat anti-mouse IgG (H + L) and Alexa Fluor 488-conjugated goat anti-rabbit IgG (H + L) [(Thermo Fisher Scientific, Waltham, MA, United States), catalog# A11004 and A27034, 1:500 dilution].

### Skeletal Preparations

Mice were euthanized at postnatal day 0, heated at 65°C in distilled water for 3 min, and de-skinned manually. Whole de-skinned embryos were stained in Alcian Blue solution (15 mg Alcian Blue dissolved in 20 ml glacial acetic acid plus 80 ml 95% ethanol) for 48–72 h, re-fixed in 100% ethanol for 24 h, and stained in Alizarin Red solution (50 mg Alizarin Red dissolved in 2% KOH) for 24 h. The samples were transferred into 50% glycerol solution for imaging and long term storage.

### Body Weight Measurement and Statistical Analysis

Mice were weighed at post-natal day 21. Body weights of 10 *Scx^+/+^*, 19 *Scx^+/–^*, and 12 *Scx*^–/–^ mice were collected and processed for statistical analysis. To compare the body weight differences among the three genotypes, we used one-way ANOVA followed by Turkey’s multiple comparison posttest. *P* value less than 0.05 was considered statistically significant and marked as ^∗^. *P* value less than 0.01 was marked as ^∗∗^, whereas *P* value less than 0.001 was marked as ^∗∗∗^ when applicable.

### ChIP-seq and Data Analysis

Three biological repeats, each containing 10 pairs of forelimbs from E13.5 *Scx*^*Flag/Flag*^ embryos, were collected for chromatin immunoprecipitation (ChIP) analysis as previously described (Xu et al., 2020). Briefly, DNA/protein complexes were extracted and incubated with the anti-FLAG antibody (Sigma, catalog# F1804). Sequencing libraries were generated using the ThruPLEX DNA sequencing kit (Rubicon Genomics). Sequencing was performed on Illumina NextSeq500. ChIP-seq reads were aligned to UCSC mouse genome 10 mm using STAR aligner (ver. 2.7.4) (Dobin et al., 2013). To match the sequencing depth across samples, aligned reads were down-sampled to 30 million. After deduplication, peaks were called for each Scx ChIP-seq dataset using HOMER (Hypergeometric Optimization of Motif EnRichment) (v4.11) (Heinz et al., 2010) against a matching input sample. To identify reproducible and unified Scx peaks, we performed second round differential analysis comparing Scx samples versus input samples anchoring on all the detected Scx peaks. First, Scx peaks from the three biological replicates were pooled and merged into a preliminary peak set. Second, read counts were measured in all the Scx ChIP samples and input samples within the peak set. Finally, exact test was performed using EdgeR (Robinson et al., 2010) comparing Scx ChIP and input samples. Final Scx peaks were defined by log_2_ fold-change (log_2_FC) over input > 2 and FDR < 0.01. *De novo* motif analysis of the Scx peaks was performed within a 200 bp window using Homer (v4.11) (Heinz et al., 2010) with default options. Peak-gene association analysis was performed utilizing the online GREAT (Genomic Regions Enrichment of Annotations Tool) program (version 4.0) (McLean et al., 2010). Gene regulatory domains utilized for region annotation were defined as minimum 5 kb upstream and 1 kb downstream of the gene transcription start site (TSS), and extended in both directions to the nearest gene’s basal domain but no more than the maximum extension of 1000 kb in one direction (‘‘Basal plus extension’’ option)^1^. The ChIP-seq raw data have been deposited into the National Center for Biotechnology Information Gene Expression Omnibus database (NCBI GEO Accession Number GSE173428).

### Fluorescence-Activating Cell Sorting (FACS)

E15.5 embryonic forelimbs were dissected from embryos of *Scx^+/–^; Scx-GFP* female crossed with *Scx^+/–^; Scx-GFP* male mice. The freshly dissected embryonic forelimbs were digested with the trypsin−EDTA solution (Invitrogen) at 37°C for 4 min. After inactivation of trypsin with DMEM containing 10% FBS, cells were dissociated by pipetting. The dissociated limb cells were suspended in PBS with 2% FBS and 10 mM EDTA, and filtered through a 40 μm nylon cell strainer (BD Falcon, 352340). GFP^+^ cells from each sample were isolated using BD FACSAria II.

### RNA−Seq and Data Analysis

RNA-seq were carried out using FACS isolated GFP^+^ cells from the forelimb tissues of three *Scx*^–/–^ embryos and three littermate controls at E15.5. Total RNAs were extracted from FACS isolated E15.5 forelimb GFP^+^ cells using the Qiagen RNeasy Micro Kit (Qiagen catalog# 74004). cDNA amplification was carried out using the Ovation RNA-Seq System V2 kit (Tecan Genomics Inc.). The sequencing libraries were made using the Nextera XT DNA Library Prep kit (Illumina), and sequenced using Illumina NovaSeq 6000 for 75 bp paired-end reads. RNA-seq reads were aligned to UCSC mouse genome 10 mm using STAR aligner (v2.7.4) (Dobin et al., 2013). Read counts for each gene were measured using FeatureCounts in the subread package (v1.6.2) (Liao et al., 2014). To avoid sex-specific bias in the analysis, genes in chromosome Y were discarded. In addition, RUVseq (RUVs, *k* = 1) (Risso et al., 2014) was applied to account for sex-specific contribution to the gene expression profiles and to identify sex-independent gene expression changes upon *Scx* deletion. Principal component analysis was applied to normalized FPKM (fragment per kilobase of transcript per million mapped reads) and log-transformed count data. Differential gene expression analysis was performed using DESeq2 (Love et al., 2014). Differentially expressed genes were identified by FDR < 0.05. Gene ontology (GO) analysis was performed using the online ToppGene tools^2^ (Chen et al., 2009). Hierarchical clustering of the differential gene expression profiles was performed using Pearson correlation coefficient of log-transformed gene expression level (FPKM) as similarity measure under Ward’s criterion. Clustering heatmap was visualized in z-score. The RNA-seq data have been deposited into the National Center for Biotechnology Information Gene Expression Omnibus database (NCBI GEO Accession Number GSE173428).

### Quantitative RT-PCR

E13.5 and E15.5 *Scx*^–/–^ mutant and wildtype control forelimb samples were collected. Total RNAs were extracted using the Qiagen RNeasy Micro Kit (Qiagen catalog# 74004), and reverse transcribed using SuperScript^TM^ III First-Strand Synthesis System (Thermo Fisher Scientific, Waltham, MA, United States catalog# 18080051). At each stage, at least 5 samples per genotype were used for quantitative RT-PCR. Relative mRNA levels were normalized to that of *Hprt* mRNAs. Student’s *t* test was used for pairwise comparison. *P* < 0.05 was considered significantly different. *P* value less than 0.05 was considered statistically significant and marked as ^∗^. *P* value less than 0.01 was marked as ^∗∗^, whereas *P* value less than 0.001 was marked as ^∗∗∗^ when applicable.

## Results

### Generation of a Novel *Scx*^*Flag*^ Mouse Line and Genome-Wide Mapping of Endogenous Scx Binding Sites in the Tendon Progenitor Cells *in vivo*

No specific antibody for the Scx protein that allows direct analysis of endogenous Scx-binding at target genes has been reported. To facilitate direct and specific analysis of endogenous Scx protein activity, we used the CRISPR/Cas9-mediated genome editing strategy (Cong et al., 2013; Scott and Hu, 2019) to insert a well-characterized 2xFLAG epitope tag at the carboxy terminus of the endogenous Scx protein in mice (Figure 1A). Detailed description of the procedures for generating the *Scx*^*Flag*^ founder mice is provided in the Materials and Methods section. The *Scx*^*Flag*/+^ founder mice were crossed to wildtype CD1 mice and the G1 *Scx*^*Flag*/+^ hemizygous progenies (Figure 1B) were sequence-verified for correct integration and germ-line transmission of the *Scx*^*Flag*^ allele as designed. The *Scx*^*Flag*/+^ mice were then intercrossed to generate *Scx*^*Flag/Flag*^ homozygous mice, which were born at expected Mendelian ratio and did not display any phenotypic difference from their hemizygous or wildtype littermates (Figures 1C–F). Immunofluorescent staining of sections of mouse embryos using an anti-FLAG antibody (Sigma, catalog# F1804) showed that the Scx-FLAG fusion protein was strongly and specifically expressed in both tendons and ligaments (Figures 1G–J), consistent with previously reported patterns of expression of endogenous *Scx* mRNAs and of the transgenic Scx-GFP reporter activity (Murchison et al., 2007; Pryce et al., 2007; Watson et al., 2009). Since the *Scx*^*Flag/Flag*^ homozygous mice do not have any phenotypic abnormality, the insertion of the FLAG epitope tag at the carboxy-terminus of the endogenous Scx protein did not affect Scx function, providing a valuable new tool for direct analysis of endogenous Scx function during development, tissue homeostasis, and injury repair.

**FIGURE 2:**
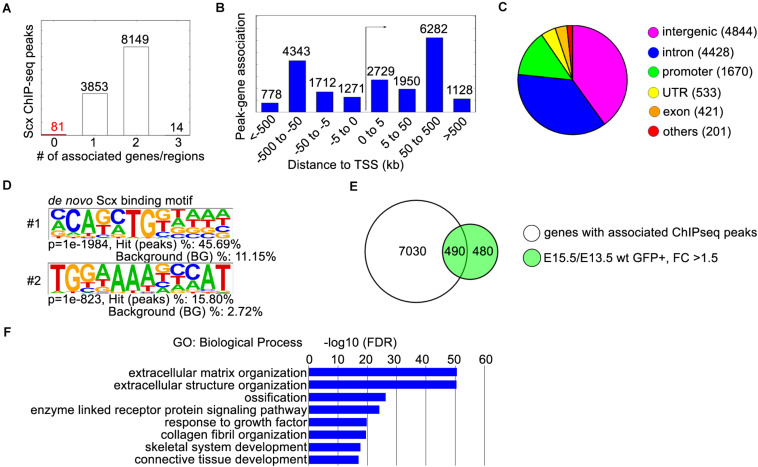
ChIP-seq analysis of E13.5 *Scx*^*Flag/Flag*^ forelimbs. **(A)** Peak-gene association analysis with the GREAT online tool (http://great.stanford.edu). The “basal plus extension” parameter was used to associate the ChIP-seq peaks to genes within 1,000 kb. The majority of peaks were associated with 1 or 2 genes. **(B)** Distribution of peak-gene associations by their distances to the transcription start site (TSS). **(C)** Distribution of ChIP-seq peaks by their locations. The majority of peaks are located in intronic (4428) or intergenic (4844) regions, while 1670 peaks were located in promoter regions. **(D)** The two most enriched Scx binding motifs from ChIP-seq peaks. **(E)** Comparison of 7520 genes with associated ChIP-seq peaks, with 970 genes that were up-regulated in GFP+ hindlimb cells from E13.5 to E15.5 by at least 1.5-fold. **(F)** Gene ontology analysis of the 490 differentially expressed genes with associated peaks from panel **(E)**. Representative GO terms are listed in the order of their *p*-values, with the most enriched GO term on top.

To investigate Scx-mediated transcriptional regulation during early tendon development, we dissected forelimbs from E13.5 *Scx*^*Flag/Flag*^ embryos and performed ChIP-seq experiments in triplicates using the anti-FLAG antibody (Sigma, catalog# F1804). Analysis of the ChIP-seq data identified 12,097 highly enriched Scx-associated chromatin regions (ChIP-seq peaks) (Figures 2A,B). About 77% (9,272 of 12,097) of the Scx ChIP-seq peaks were located in intronic or intergenic regions associated with one or two genes in the immediate vicinity within 500 kb from the gene’s transcription start site (TSS), whereas only about 14% (1,670 of 12,097) of the Scx-binding sites were located within gene promoter regions (Figures 2A–C). *De novo* motif analysis revealed that the most enriched endogenous Scx-binding domain contains a core sequence C-A-G/T-A/C-T-G (Figure 2D), which was present in >45% of the ChIP-seq peak regions and matches the Scx-binding core sequence previously determined by electrophoresis mobility shift assays (Shukunami et al., 2018). The second most enriched motif, which is present in 15.8% of the ChIP-seq peaks, contains a core sequence that matches the consensus binding motif of the Nfat family transcription factors, TGGAAA (Yu et al., 2015; Klein-Hessling et al., 2017; Figure 2D). It has been reported that Scx and Nfatc4, a member of the Nfat family, act cooperatively to regulate the transcription of *Col1a1* in tendon fibroblast cells (Lejard et al., 2007). The significant enrichment of the Nfat binding motif in the Scx ChIP-seq peaks indicate that Scx and Nfat family proteins act together to regulate expression of many genes during tendon development.

Genomic Regions Enrichment of Annotations Tool analysis of the ChIP-seq data recovered 7,520 genes associated with the Scx-binding genomic regions. To gain insight into Scx-mediated transcriptional regulation during early tendon development, we compared the ChIP-seq data with the previously reported RNA-seq data analyzing transcriptome profiles of developing mouse hindlimb tendons (Liu et al., 2015). We found that high quality Scx ChIP-seq peaks were detected in 490 of the 970 genes whose expression was upregulated by more than 1.5-fold in Scx-GFP^+^ cells during early tendon cell differentiation in the mouse hindlimb from E13.5 to E15.5 (Figure 2E). GO analysis showed that this group is highly enriched with genes playing roles in “extracellular matrix organization,” “collagen fibril organization,” and “connective tissue development” (Figure 2F), consistent with a major role of Scx in regulating tendon formation.

### Characterization of a New *Scx* Mutant Mouse Resource

**FIGURE 3:**
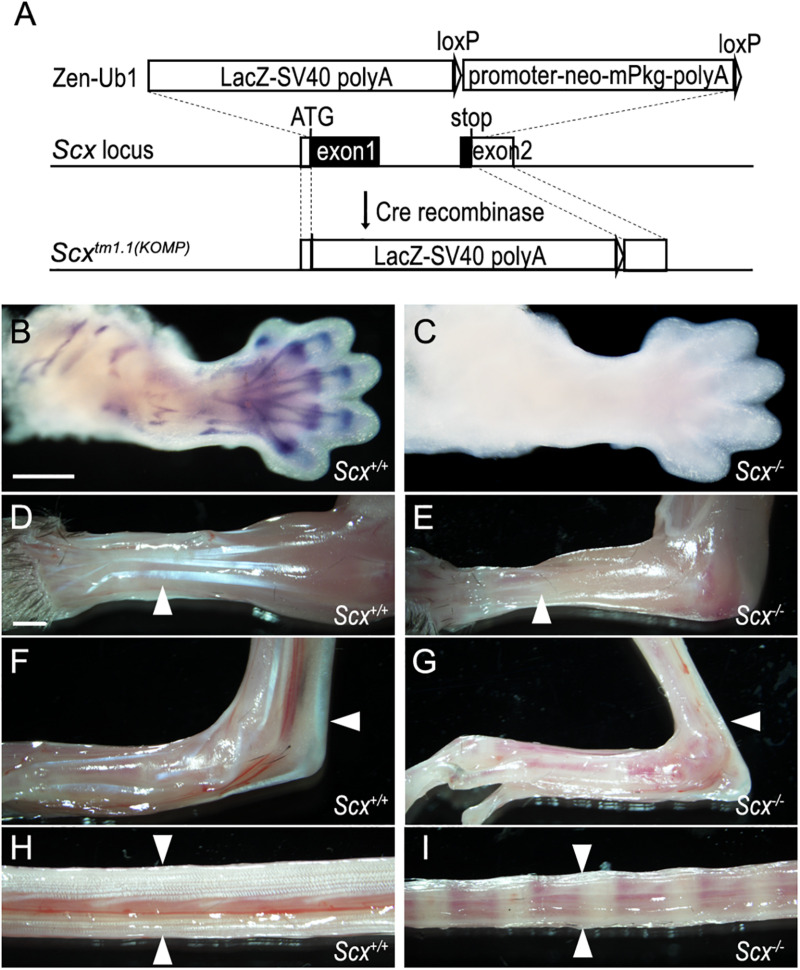
Characterization of a *Scx* null mutant mouse strain. **(A)** Schematic diagram and strategy of generating *Scx* null mutant allele. A VelociGene ZEN-Ub1 LacZ reporter cassette was inserted after the endogenous *Scx* ATG codon, by homologous recombination, and replaced the entire *Scx* coding sequence plus a 56 bp 3′ UTR sequence. **(B,C)** *In situ* hybridization on E13.5 *Scx*^–/–^ mutant and control forelimbs, with an anti-sense RNA probe against the protein coding region of the *Scx* mRNAs. **(D–I)** Whole mount view of tendons in forelimb **(D,E)**, hindlimb **(F,G)**, and tail **(H,I)** from *Scx*^–/–^ mutant **(E,G,I)** and control littermates **(D,F,H)** at P21. White arrowheads point to extensor digitorium communis in panels **(D,E)**, Achilles tendons in panels **(F,G)**, and tail tendons in panels **(H,I)**. Scale bar is 500 μm in panel **(B)** and 1000 μm in panel **(D)**.

Whereas several distinct *Scx* mutant mouse lines have been reported (Murchison et al., 2007; Yoshimoto et al., 2017; Shukunami et al., 2018), none of these are available to the wide biomedical research community through the Mutant Mouse Resource Centers. On the other hand, the United States National Institutes of Health-funded Knock-out Mouse Project (KOMP) Consortium has generated a *Scx*-knockout mouse line [C57BL/6N-*Scx^*tm1.1(KOMP)Vlcg*^/MbpMmucd*] and made available through the Mouse Biology Program at the University of California Davis, but the tendon developmental defects in mice homozygous for this particular *Scx* knockout allele has not been described. We obtained the C57BL/6N-*Scx^*tm1.1(KOMP)Vlcg*^/MbpMmucd* mouse line and established a breeding colony. The *Scx*^*tm1.1(KOMP)*^ allele (abbreviated as *Scx*^–^ in the rest of the report) contains an insertion of the VelociGene ZEN-Ub1 LacZ reporter cassette replacing the entire protein-coding sequences from the translation start site in Exon-1 to 56 bp 3′ to the translation STOP codon in Exon-2 of the *Scx* gene (Figure 3A). We confirmed the structure and correct integration of the *lacZ* reporter cassette at the *Scx* locus through Sanger sequencing of genomic PCR products. However, no specific beta-galactosidase reporter activity was detected in developing tendon tissues in the heterozygous and homozygous mutant mouse embryos, likely due to reporter gene silencing described previously in multiple other KOMP mouse lines (Kirov et al., 2015). To confirm that Scx function is lost in the *Scx*^–/–^ embryos, we carried out *in situ* hybridization assay using a probe specific for the *Scx* coding sequence and found that *Scx* mRNA expression was completely absent in the *Scx*^–/–^ embryos (Figures 3B,C).

We further characterized the phenotypes of the *Scx*^–/–^ mutant mice. *Scx*^–/–^ mice were born at Mendelian ratio, but with reduced body size and severely impaired limbs, with the autopod of both fore- and hind-limbs locked in a dorsal flexure (Supplementary Figure 1). All neonatal *Scx*^–/–^ mutant mice exhibited severe hypoplasia of major limb and tail tendons (Figures 3D–I). Analysis of skeletal preparations showed that *Scx*^–/–^ mutants lacked the deltoid tuberosity of the humerus in the forelimb and exhibited reduced size of the patella and the entheseal cartilage of the calcaneus in the hindlimb (Supplementary Figures 2A–D). In addition, the transverse processes of the lumbar vertebrae were reduced in the *Scx*^–/–^ mutants (Supplementary Figures 2E, F). Using a previously reported Scx-GFP transgenic reporter (Pryce et al., 2007), we found that tendon differentiation and condensation in the developing limbs were disrupted by E15.5 in the homozygous mutant embryos (Supplementary Figure 3). The tendon and skeletal defects in these *Scx*^–/–^ mice are similar to those reported previously in two other independent *Scx* mutant mouse lines (Murchison et al., 2007; Shukunami et al., 2018). Thus, this KOMP-generated *Scx* knockout mouse line is a valuable resource for the biomedical research community for further studies of Scx function *in vivo*.

### Analysis of Scx-Dependent Transcriptome Expression Profiles During Early Tendon Cell Differentiation *in vivo*

**FIGURE 4:**
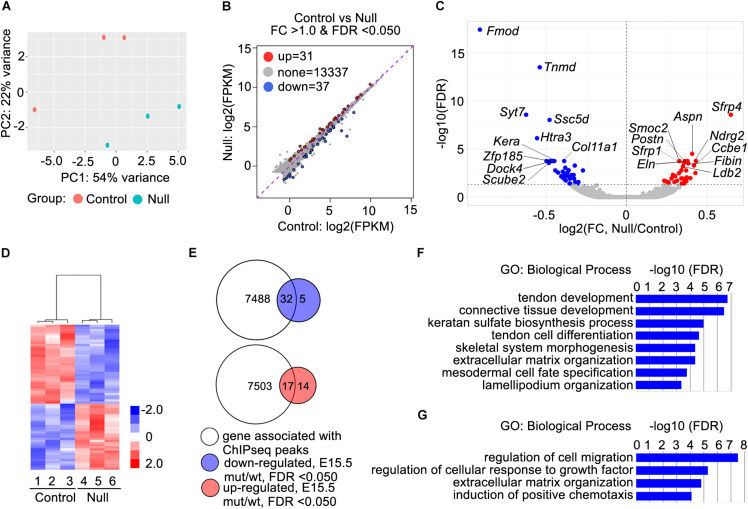
RNA-seq data analysis and identification of candidate Scx direct target genes. **(A)** Principal component plot displaying the three *Scx*^–/–^ and three control samples along PC1 and PC2, which account for 54 and 22%, respectively, of the variability within the RNA-seq dataset. **(B)** MA plot displaying differential expression profiles of the *Scx*^–/–^ and control samples. Up-regulated genes are marked in red whereas down-regulated genes are marked in blue. **(C)** Volcano plot displaying the log_2_ fold change against the log_10_ value of the FDR adjusted *P* value. Up-regulated genes are shown in red while down-regulated genes shown in blue. Top 10 down-regulated and top 10 upregulated genes are marked by with symbol. **(D)** Heatmap showing hierarchical clustering on the 68 differentially expressed genes. The z-score scale bar represents relative expression+/- 2SD from the mean. **(E)** Comparisons of 7520 genes with associated Scx binding peaks with 37 down-regulated genes in *Scx*^–/–^ samples at E15.5 and 31 up-regulated genes in *Scx*^–/–^ samples at E15.5 **(B)**. **(F,G)** Gene ontology analyses of 32 down-regulated genes associated with Scx-binding peaks and 17 up-regulated genes associated with Scx-binding peaks, respectively. Representative GO terms are listed in the order of their *P* values, with the most enriched GO term on top.

We crossed the *Scx-GFP* transgenic reporter (Pryce et al., 2007) into the new *Scx^+/–^* mouse line and then intercrossed *Scx^+/–^; Scx-GFP* mice for analysis of transcriptomic effects of Scx during early tendon cell differentiation. Scx-GFP^+^ cells were isolated by FACS from freshly dissected forelimb tissues from E15.5 wildtype, *Scx^+/–^*, and *Scx*^–/–^ mutant embryos for RNA-seq analysis. Analysis of the RNA-seq results from three *Scx*^–/–^ embryos and three control littermates identified 68 genes that exhibited significant changes in expression levels (FDR < 0.05) in the forelimb Scx-GFP^+^ cells in E15.5 *Scx*^–/–^ embryos compared with their control littermates (Figures 4A–D). Comparison of the Scx-dependent differentially expressed genes with the Scx ChIP-seq data revealed that 32 of the significantly down-regulated genes were associated with Scx occupancy in the tendon progenitor cells (Figure 4E and Table 1). These genes are likely critical direct target genes mediating Scx function in tendon formation and early tendon cell differentiation. Indeed, GO analysis of this gene group showed that “tendon development,” “tendon cell differentiation,” “keratan sulfate biosynthesis,” and “ECM organization” are among the most significantly enriched biological processes (Figure 4F). The most significantly down-regulated Scx target genes in the E15.5 *Scx*^–/–^ forelimb tendon cells include *Fmod*, *Tnmd*, *Kera*, and *Col11a1* (Figure 4C and Table 1), of which each plays important roles in tendon cell differentiation and/or collagen fibrillogenesis (Svensson et al., 1999; Pellegata et al., 2000; Docheva et al., 2005; Kilts et al., 2009; Wenstrup et al., 2011; Sun et al., 2020) but only *Tnmd* has previously been identified as a Scx transcriptional target gene (Li et al., 2015; Shukunami et al., 2018). The Scx-dependent target genes also include three genes encoding transcription factors, *Mkx*, *Six2*, and *Eya1* (Table 1). Mkx function is required for tendon fibril growth and tendon homeostasis (Ito et al., 2010; Liu et al., 2010, 2019; Suzuki et al., 2016).

**TABLE 1:** Genes with associated Scx-binding peaks and down-regulated in *Scx*^–/–^ tendon cells at E15.

Gene symbol	Scx-binding peaks (numbers indicate distance to TSS, in bp)							
*Adgrg2*	−184004							
*Aqp1*	−90524*	−41742	−3597	−3169	+2103			
*C1qtnf3*	−229323*	−79453	−75909	−23517	−11848	−3835*	−623*	
*Ccdc85a*	+114267	+118157						
*Ccdc88a*	−13517	−2700	−1					
*Chst5*	+5231	+5847						
*Cilp2*	+9479							
*Col11a1*	−170353	−98636	+34903	+35353				
*Col22a1*	+106886							
*Cyr61*	+931							
*Dock4*	+210357	+248137	+250413	+256430	+256533	+295104	+342939	+343124
*Enpp2*	+114774							
*Eya1*	−375541	−112658	−47591	+52976	+165834	+173401	+378637	+382083
*Fat3*	−425380	−329482	−140752	−895	+33046	+230423	+246601	+348404*
*Fmod*	−95614	−58501	−23204	−26				
*Htra3*	+57656	+70290*						
*Kera*	+5138	+12293	+17683	+19969				
*Mkx*	−231675*	−227917*	−83259	−33399				
*Mtcl1*	+20857	+32469	+84844	+214107				
*Naalad2*	+4887							
*Olfml2b*	−38976	−5227	+7772	+11134	+57278	+74224		
*Plch1*	+27008	+268469	+357217					
*Rflnb*	−132608*	−97812*	−71599	−15311	−14423	−14271	−55	
*Scube2*	+14689	+30597						
*Six2*	−247206	−242150	−218515	−205193	−193527	−173342	−164957	−135585
*Ssc5d*	−776							
*St8sia1*	+18010	+26582	+26704	+149719*				
*Syt7*	−69362	−7436	+14213	+39584*				
*Thbs4*	−37977	−33745	+62949	+155236*				
*Tmem44*	+14520	+71219						
*Tnmd*	−90582	−20742						
*Zfp185*	−423							

*Peak located in neighboring gene.

Analysis of RNA-seq data also revealed 31 genes that were significantly up-regulated in the Scx-GFP^+^ forelimb cells of E15.5 *Scx*^–/–^ embryos compared with control littermates (Figures 4B–D). Among these, 17 genes were associated with Scx ChIP-seq peaks in the tendon progenitor cells (Figure 4E and Table 2). GO analysis of this group of genes showed that many of these, including *Ccbe1*, *Cxcl12*, *Epha3*, *Fgf10*, *Igf1*, *Ldb2*, *Postn*, *Smoc2*, and *Surf1*, are involved in “regulation of cell migration” (Figure 4G). In addition, *Surf1*, *Postn*, *Smoc2, Eln*, and *Nid2* are involved in regulation of “extracellular matrix organization,” whereas *Cxcl12* and *Fgf10* have been implicated in “induction of chemotaxis” (Figure 4G and Table 2). Taken together, Scx regulates early tendon cell differentiation by controlling the expression of multiple ECM components as well as signaling pathways controlling cell behavior.

**TABLE 2:** Genes with associated Scx-binding peaks and upregulated in *Scx*^–/–^ tendon cells at E15.5.

Gene symbol	Scx-binding peaks (numbers indicate distance to TSS, in bp)							
*Adgrb3*	−746	+600918*						
*Aspn*	−37162*	+1353	+3527					
*Ccbe1*	−112895	+57507*	+192663*	+210538*				
*Cxcl12*	−311414	−222186	−200466	−83159	−63138	+18174	+139363	+248065
*Dio2*	+329510							
*Eln*	−153983*	−148138	−70385					
*Epha3*	+7097	+244725	+250929	+307693	+576357	+747465	+779839	+779948
*Fgf10*	−237483	+195887						
*Fibin*	−50132	−50030	−48436	−29864	+124	+267	+9459	+13190
*Igf1*	−206628	−183042	−182419	−148	+1161	+187057	+188639	
*Ldb2*	−322438*	−123018	−15277	+214796				
*Lingo2*	−801973*	−15799	+145957					
*Nid2*	+66867*							
*Postn*	−10601	+127772	+129401	+226369	+297040	+304008		
*Prg4*	−339455*	−294031*	−251594	−149528	−106405	−23787		
*Smoc2*	−48448	+34969	+91272*	+217732	+231118	+327987	+385193*	
*Sulf1*	−513097	−254232	−111144	+194				

*Peak located in neighboring gene.

### Multiple Scx Target Genes Exhibited Specific Scx-Dependent Expression During Tendon Differentiation

**FIGURE 5:**
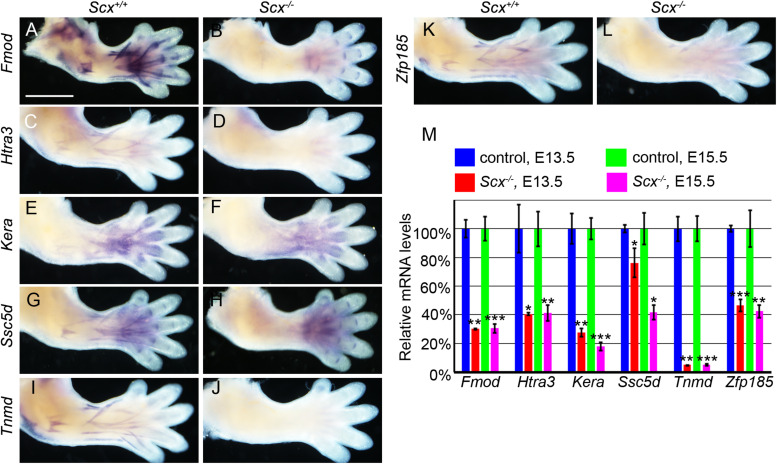
Characterization of candidate genes from ChIP-seq and RNA-seq. **(A–L)** Whole mount *in situ* hybridization of six candidate genes in E14.5 *Scx*^–/–^ mutant **(B,D,F,H,J,L)** and control **(A,C,E,G,I,K)** forelimbs. Expression of *Fmod, Kera, Ssc5d*, and *Zfp185* was dramatically reduced in *Scx*^–/–^ mutant limbs **(B,F,H,L)**, while expression of *Htra3* and *Tnmd* were nearly completely absent in *Scx*^–/–^ mutant **(D,J)**. Expression of *Fmod* and *Ssc5d* persisted in digit ligaments in the mutant limbs **(B,H)**. **(M)** Quantitative RT-PCR analysis of expression of the six candidate genes in *Scx*^–/–^ mutant and control forelimb samples at E13.5 and E15.5. All genes were significantly reduced in *Scx*^–/–^ mutants at both stages, compared with control littermates. *p < 0.05, **p < 0.01, ***p < 0.001.

**FIGURE 6:**
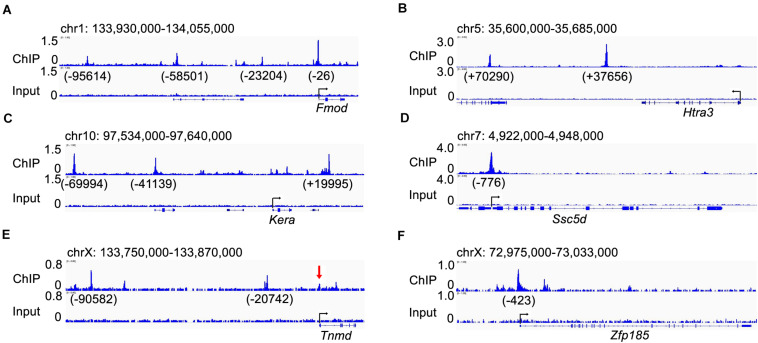
Visualization of the Scx-binding peaks associated with the candidate Scx direct target genes. **(A–F)** genome browser views of genomic regions containing, *Fmod* **(A)**, *Htra3* **(B)**, *Kera* **(C)**, *Ssc5d* **(D)**, *Tnmd* **(E)**, and *Zfp185* **(F)** genes. Numbers under each peak indicate the distance to the transcription start site (TSS) the marked gene. The red arrow in panel **(E)** point to a Scx-binding peak at the Tnmd gene promoter region that is recognizable upon careful examination but was below the FDR < 0.01 threshold for identifying genome-wide Scx binding sites in our ChIP-seq data analysis.

We further analyzed the patterns of expression of the top 10 down-regulated genes in the E15.5 *Scx*^–/–^ embryos from the RNA-seq data. Whole mount *in situ* hybridization analyses showed that the *Fmod, Htra3, Kera, Ssc5d, Tnmd*, and *Zfp185* genes each exhibited highly specific patterns of expression in the developing tendon tissues in E14.5 wildtype embryos and their expression in the developing limb tendons was dramatically reduced in the *Scx*^–/–^ littermates (Figures 5A–L). Furthermore, quantitative real-time RT-PCR analysis revealed that expression of each of these genes was already significantly reduced in the Scx-GFP^+^ forelimb mesenchyme cells in *Scx*^–/–^ embryos by E13.5, in comparison with their control littermates (Figure 5M). Among those, *Fmod* and *Tnmd* have been reported to play important roles in tendon development whereas *Kera* plays crucial roles in other connective tissues including the cornea (Svensson et al., 1999; Pellegata et al., 2000; Docheva et al., 2005; Kilts et al., 2009). *Htra3* and *Zfp185* exhibited specific expression in the developing tendons, similar to *Tnmd*, whereas *Ssc5d* exhibited a similar pattern of expression as *Fmod* in both tendon cells and in joint cells (Figure 5). Further *in situ* hybridization analysis of serial transverse sections through the digit, metacarpal, and zeugopod regions of the E15.5 forelimb samples confirmed the specificity of expression of *Htra3*, *Zfp185*, and *Tnmd* throughout the differentiating tendon cells (Supplementary Figure 4). Examination of the Scx ChIP-seq profiles associated with these genes showed high quality enrichment of Scx binding at the basal promoter regions of *Fmod*, *Ssc5d*, and *Zfp185* whereas Scx binding was also specifically enriched at distal regulatory elements associated with *Fmod*, *Kera*, *Htra3*, and *Tnmd* (Figure 6). Whereas the roles of *Htra3*, *Ssc5d*, and *Zfp185* in tendon development remain unknown, our finding that they are direct Scx targets that exhibit Scx-dependent activation during early tendon differentiation suggests that they play crucial roles downstream of Scx in tendon development.

## Discussion

Previous genetic studies have identified Scx as the most crucial transcriptional regulator of tendon development as well as tendon homeostasis and repair (Murchison et al., 2007; Yoshimoto et al., 2017; Shukunami et al., 2018; Gumucio et al., 2020). Mice lacking Scx exhibited loss or severe disruption of force-transmitting tendons throughout the body as well as defects in entheseal development (Murchison et al., 2007; Killian and Thomopoulos, 2016; Yoshimoto et al., 2017). However, the molecular mechanisms mediating Scx function in tendon development is not well understood and very few Scx target genes in tendon cells have been identified. The lack of large scale identification of direct target genes of Scx in tendon development is largely due to the lack of a reliable specific antibody for mapping genome-wide Scx binding in tendon cells *in vivo*. Scx belongs to the class II bHLH family of transcription factors, of which many have been shown to bind to an E-box (CANNTG) motif (Cserjesi et al., 1995; Massari and Murre, 2000). However, this short redundant sequence can be found throughout the genome. Thus, previous studies of Scx-mediated transcriptional regulation of putative target genes primarily relied on *in vitro* biochemical assays including electrophoresis mobility shift assay of recombinant Scx protein binding to E-box containing promoter sequences and promoter reporter assays in cell transfection studies (Lejard et al., 2007; Shukunami et al., 2018; Paterson et al., 2020), and more recently ChIP-qPCR assays of candidate promoter regions (Paterson et al., 2020). In this study, we have generated *Scx*^*Flag*^ mice and demonstrate that this new mouse line enables direct analysis of endogenous Scx protein function in the normal developmental and physiological context. Whereas we have used this mouse line for genome-wide mapping of Scx binding sites in early developing limb tendon tissues, this mouse line will provide a valuable tool for direct analysis of Scx function in many developmental and disease processes where Scx plays a role.

Our ChIP-seq analysis identified 12,097 high quality Scx binding sites in the E13.5 mouse forelimb, with the most highly enriched *de novo* motif identified as 5′ CAG/TA/CTG 3′. Whereas previous *in vitro* studies have shown Scx binding to various E-boxes including CACGTG in the *Col1a1* promoter (Lejard et al., 2007), CAGGTG in the *Col2a1* promoter (Furumatsu et al., 2010), and CAAATG and CAGATG in the *Tnmd* promoter (Li et al., 2015), direct comparative EMSA analysis of Scx binding to five distinct E-box containing elements from the mouse *Tnmd* promoter region showed that Scx preferentially bound to CAGATG and CATCTG, but not the others (Shukunami et al., 2018). Thus, the consensus Scx binding motif from our ChIP-seq data matches perfectly with the EMSA-determined preferential Scx binding motif, which affirms our genome-wide mapped Scx-binding sites. Whereas several *in vitro* studies have repeatedly demonstrated Scx binding to the *Tnmd* promoter regions and Scx activated expression of *Tnmd* promoter-reporter constructs in co-transfected cells (Li et al., 2015; Shukunami et al., 2018), our ChIP-seq results showed that endogenous Scx binding was primarily enriched at two upstream locations at about 20 and 90 kb, respectively, from the *Tnmd* TSS (Figure 6E). A minor Scx binding peak was detected at the *Tnmd* basal promoter region (Figure 6E), which is enriched over 4-fold over the input but the FDR value, at 0.025, was below the stringent threshold (4-fold enrichment over input and FDR < 0.01) used for identifying the genome-wide Scx binding peaks in our analysis. It is possible that Scx binding to both the distal enhancers and the promoter region to synergistically activate *Tnmd* gene expression in tenocytes.

Whereas previous studies have shown that activation of *Tnmd* expression during tendon development depends on Scx function and that Tnmd plays crucial roles in tenocyte proliferation and maturation, *Tnmd* null mice exhibited a much milder tendon phenotype than *Scx*^–/–^ mice (Docheva et al., 2005; Murchison et al., 2007; Yoshimoto et al., 2017; Shukunami et al., 2018). Our ChIP-seq and RNA-seq results demonstrate that Scx directly regulates expression of a large number of genes during early tendon development, including activation of expression of many tendon cell-specific genes. In particular, we validated *Fmod, Htra3, Ssc5d*, and *Zfp185* as new direct target genes that dependent on Scx for their expression in the differentiating tendon cells. *Fmod*-deficient mice exhibited defects in tendon collagen fibrillogenesis (Chakravarti, 2002), but the roles of *Htra3*, *Ssc5d*, and *Zfp185* in tendon development are unknown. *Htra3* encodes a serine peptidase that cleaves proteoglycans, thus may function in ECM remodeling (Nie et al., 2003; Glaza et al., 2015). *Ssc5d* (*scavenger receptor cysteine rich family member with 5 domains*) has been implicated in playing a role at the interface between adaptive and innate immunity and in placental function (Goncalves et al., 2009). *Zfp185* encodes LIM domain type zinc finger protein (Heiss et al., 1997; Zhang et al., 2016). Whereas the Zfp185 protein was originally hypothesized to reside in the cell nucleus, subsequent *in vitro* cell biological studies suggested that Zfp185 binds to F-actin and may be involved in modulating dynamics of actin filaments (Wang et al., 2008; Zhang et al., 2016). Thus, these gene products have diverse cellular functions and loss of their expression during early tendon cell differentiation in the *Scx*^–/–^ embryos likely contributed to the severe disruption of tendon condensation phenotype.

Whereas our ChIP-seq analysis identified more than 12,000 high quality Scx-binding regions associated with over 7,500 genes in the embryonic forelimb tissues, our RNA-seq analysis uncovered a relatively small number of genes that exhibited significant differential expression in the Scx-GFP^+^ forelimb tendon cells between E15.5 *Scx*^–/–^ and control littermates. These results suggest that Scx participates in the regulation of a large number of genes during early tendon development, but other factors likely partly compensate for Scx function in the *Scx*^–/–^ mice. Nevertheless, the differential gene expression profiles uncovered by our RNA-seq data provide new insights into the molecular mechanisms underlying Scx-mediated regulation of tendon formation. It has been shown that the tendon microfibrils are highly disorganized in the *Scx*^–/–^ mouse embryos (Murchison et al., 2007). We found that expression of *Col11a1* was among the most down-regulated genes in the E15.5 *Scx*^–/–^ forelimb tendon cells. Recent studies have shown that ColXI, the gene product of *Col11a1*, plays an essential role in tendon fibril assembly and organization (Wenstrup et al., 2011; Sun et al., 2020). Mice with tendon-specific disruption of *Col11a1* in the Scx-expressing lineages exhibited abnormal tendon fibril structure, smaller fibril diameter and disrupted fibril alignment (Sun et al., 2020). Thus, activation of *Col11a1* expression in the differentiating tenocytes is likely an important part of Scx function in tendon formation. In addition to regulating tendon ECM organization and fibrillogenesis, Scx plays a crucial role in regulating tendon cell morphology and organization (Murchison et al., 2007). Whereas wildtype tenocytes develop a complex network of cytoplasmic extensions that engulf the collagen fibril bundles during tendon fibrillogenesis, the tenocytes in *Scx*^–/–^ mouse embryos exhibited much reduced and less complex cytoplasmic extensions (Murchison et al., 2007). We found that *Dock4*, encoding a key regulator of filapodia and lamellipodia protrusions (Hiramoto et al., 2006; Abraham et al., 2015), was among the top downregulated genes in the E15.5 tendon cells. As tenocyte cytoplasmic extensions likely also involve spatially regulated changes of the actin cytoskeleton, our finding of significant down-regulation of *Zfp185*, *Ccdc85a* and *Ccdc88a*, which all encode actin binding proteins, in the tendon cells in E15.5 *Scx*^–/–^ embryos suggests that Scx regulates expression of multiple cytoplasmic proteins to control tenocyte morphology and organization. Moreover, to maintain the stretched tenocyte morphology with a complex network of cytoplasmic extensions during tendon growth and elongation likely requires cell membrane repair mechanisms to maintain plasma membrane integrity. In *Scx*^–/–^ mice tendon rudiments were detected in the embryonic tail but tail tendon was completely absent in 2-week-old mutant mice due to increased apoptosis (Murchison et al., 2007). Among the most significantly downregulated genes in the *Scx*^–/–^ tendon cells in our RNA-seq data is *Syt7* (Figure 4C), which encodes a transmembrane protein with a crucial role in maintenance of plasma membrane integrity and repair via regulating lysosomal exocytosis (Chakrabarti et al., 2003). Further studies will be needed to investigate whether Syt7 plays an important role in Scx-mediated tenocyte differentiation and maintenance. Furthermore, it has been shown that Scx function is required for proper organization of tendon sheath such as the endotenon and that Epha4-expressing endotenon cells were detected intermixed with tenocytes in the limb tendons in *Scx*^–/–^ embryos (Murchison et al., 2007). Our RNA-seq data showed that expression of *Epha3* was significantly increased in the limb tendon cell in E15.5 *Scx*^–/–^ embryos. It is possible that the increased *Epha3* expression in the tenocytes resulted in disruption of the Eph-Efn signaling involved in regulation of tendon sheath formation.

Whereas *Scx* is expressed in all tendon progenitor cells and throughout of tendon development (Schweitzer et al., 2001; Brent et al., 2003), only a subset of tendon tissues were significantly disrupted in *Scx*^–/–^ mice (Murchison et al., 2007). Huang et al. (2019) demonstrated that Scx function is required for recruitment of mesenchymal progenitor cells into the initially formed tendon rudiments during the growth and elongation of the limbs and tail (Huang et al., 2019). Remarkably, lineage-specific genetic analysis and cell transplantation assays demonstrated that Scx function is exclusively required in the recruited mesenchymal cells, but not in the recruiting tendon, for the recruitment and integration of the mesenchymal progenitor cells during tendon elongation (Huang et al., 2019). The molecular mechanism acting downstream of Scx in mediating the tendon cell recruitment and elongation is currently unknown. Although our RNA-seq data show significant changes in expression of several signaling molecules involved in regulation of cell migration and/or chemotaxis in the *Scx*^–/–^ Scx-GFP^+^ forelimb cells, it is not clear whether and how some of these molecules may mediate Scx function in tendon cell recruitment. Further analysis of the detailed spatiotemporal patterns of expression of these genes during tendon elongation and/or single-cell transcriptomic profiling combined with lineage-specific functional studies will help resolve the underlying molecular mechanism.

In a recent report, Gumucio et al. (2020) performed RNA-seq analysis to identify genes and signaling pathways that respond differently to mechanical overloads in the plantaris tendons in adult mice due to conditional inactivation of *Scx* (Gumucio et al., 2020). We compared our RNA-seq results from embryonic tendon cells with their RNA-seq results from the adult tenocytes and found that there were a number of overlapping genes in both the down-regulated and up-regulated groups of Scx-dependent differentially expressed genes. In particular, the *Fmod*, *Kera*, *Ssc5d*, *Tnmd*, and *Zfp185* genes were significantly down-regulated in the adult *Scx*-deficient mutant tendon tissues. These genes likely play important roles in tendon cell differentiation at both embryonic and adult stages. Thus, further elucidation of the molecular mechanisms of tendon development will facilitate investigation of adult tendon cell behaviors during injuries and regeneration and contribute ultimately to improvement in strategies for tendon therapies. In this regard, our ChIP-seq and RNA-seq datasets provide a rich resource for aiding the design of new studies of the molecular and cellular mechanisms of tendon development and repair.

## Data Availability Statement

ChIP-seq and RNA-seq data was deposited in the NCBI GEO database under the accession number(s) GSE173428.

## Ethics Statement

The animal study was reviewed and approved by Cincinnati Children’s Research Foundation Institutional Animal Care and Use Committee (IACUC).

## Author Contributions

HL, YL, and RJ conceptualized and designed the research. HWL helped revise the research design and data analysis during the manuscript revision. HL and JX performed the research. HL, JX, YL, HWL, and RJ analyzed the data and critically revised the manuscript. HL and RJ wrote the manuscript. All authors approved the final manuscript and agreed to be accountable for all aspects of the work in ensuring that questions related to the accuracy or integrity of any part of the work are appropriately investigated and resolved.

## Conflict of Interest

The authors declare that the research was conducted in the absence of any commercial or financial relationships that could be construed as a potential conflict of interest.

## Footnotes

1 http://great.stanford.edu

2 https://toppgene.cchmc.org
