# Supplementary material for: The Scleraxis Transcription Factor Directly Regulates Multiple Distinct Molecular and Cellular Processes During Early Tendon Cell Differentiation
Source: Front Cell Dev Biol. 2021 Jun 3;9:654397. doi: 10.3389/fcell.2021.654397 (PMC8211106; doi:10.3389/fcell.2021.654397)
Supplement: Supplementary file 1 [file Data_Sheet_1.PDF]

Supplementary figures for Liu et al., “**The Scleraxis transcription factor directly regulates multiple distinct molecular and cellular processes during early tendon cell differentiation”**

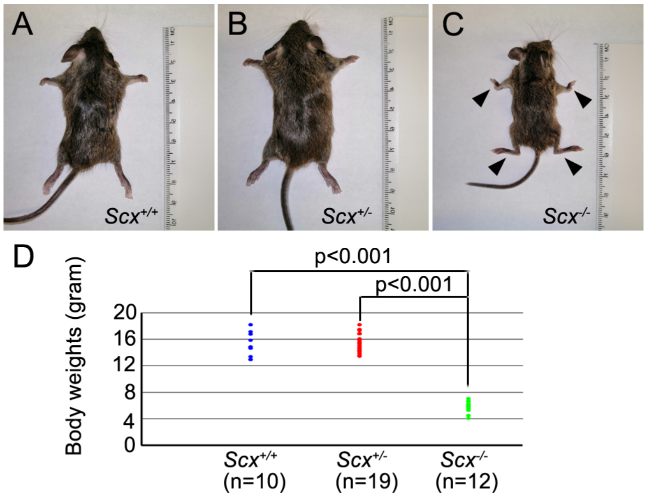

Supplementary Figure S1. *Scx*$^{-/-}$ mice exhibit postnatal growth retardation and abnormal limbs. (A-C) Dorsal view of wildtype (A), *Scx*$^{+/-}$ (B), and *Scx*$^{-/-}$ littermates at postnatal day 21. Arrowheads point to the aberrantly locked autopod posture. (D) Body weight measurements of the wildtype, *Scx*$^{+/-}$, and *Scx*$^{-/-}$ mice at postnatal day 21.

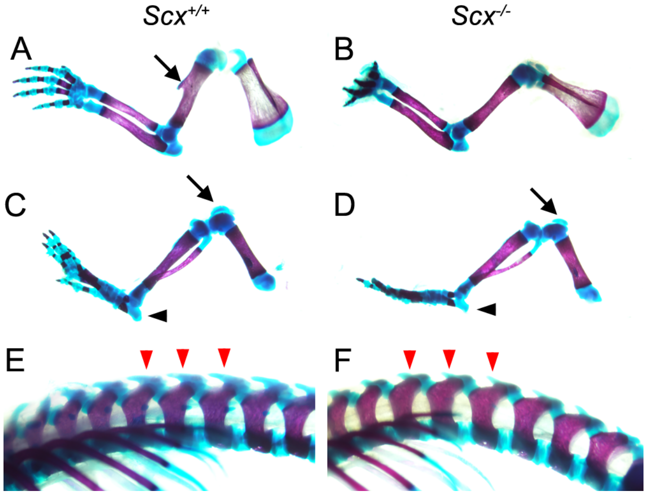

Supplementary Figure S2. *Scx*$^{-/-}$ mice exhibit defective tendon-bone attachment sites. (A, B) *Scx*$^{-/-}$ embryos lack the deltoid tuberosity (arrow) of the humerus compared with the wildtype littermates.

(C, D) *Scx*$^{-/-}$ embryos exhibited reduced size of the patella (arrow) and the entheseal cartilage of the calcaneus (arrowhead) in the hind limbs compared with the wildtype littermates.

(E, F) *Scx*$^{-/-}$ embryos exhibited reduced vertical processes (red arrowheads) of the lumbar vertebrae compared with the wildtype littermates.

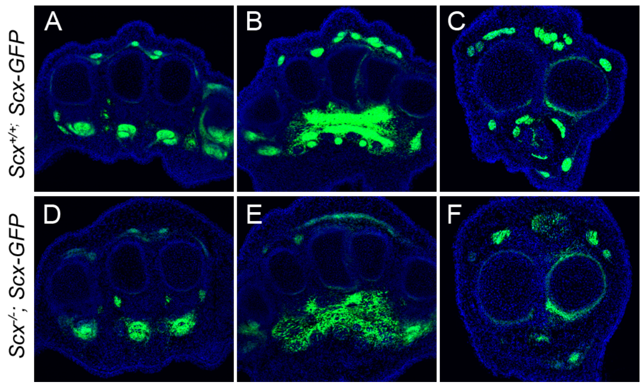

Supplementary Figure S3. Scx-GFP expression in *Scx*$^{-/-}$ mutant and controls.
(A-F) Cross cryo-section of E15.5 *Scx*$^{-/-}$ mutant and control forelimbs, from distal (A-D), middle (B, E) to proximal (C, F). Scx-GFP (green) is still expressed in mutant (D, E, F), but in a weaker and disorganized pattern, compared with control (A, B, C).

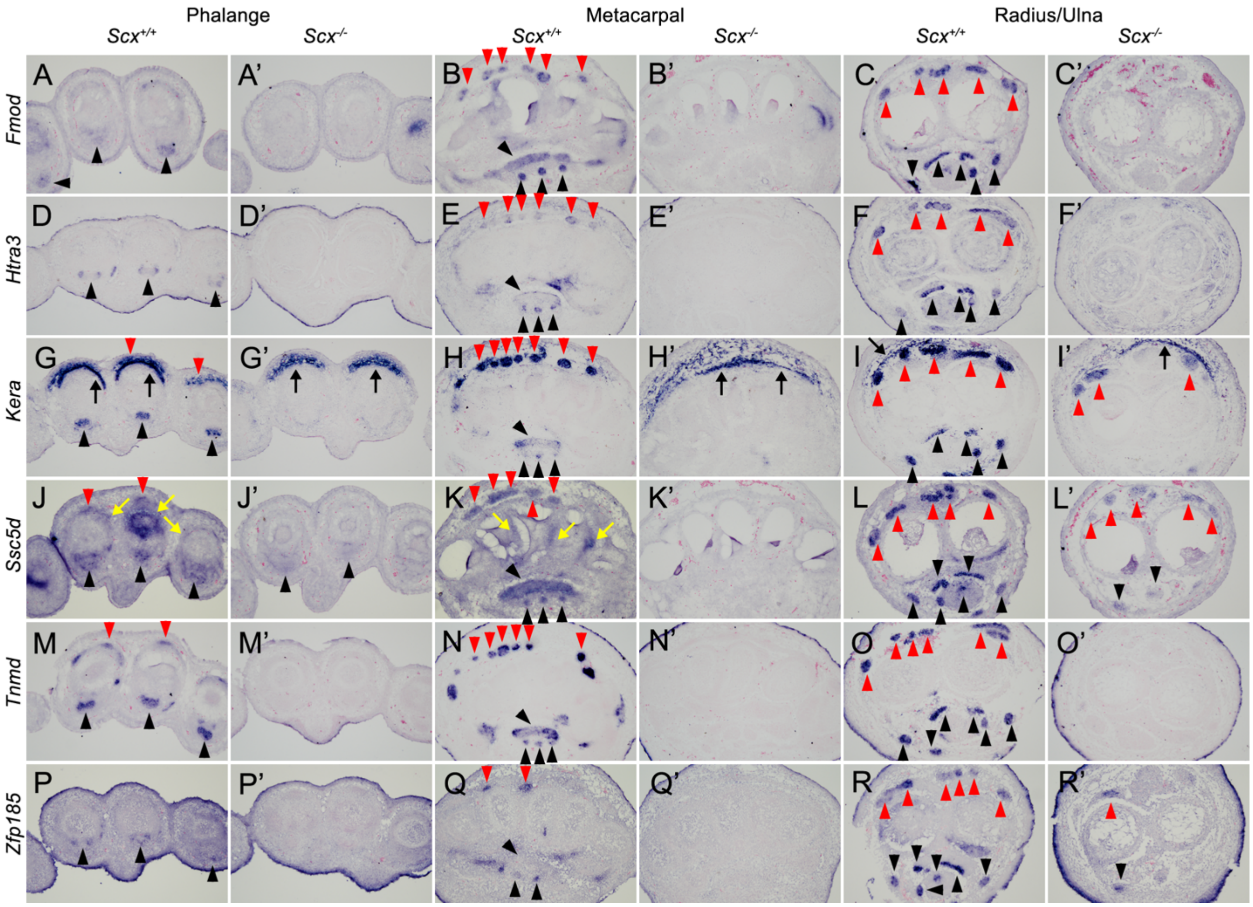

Supplementary Figure S4. Comparison of expression of *Fmod*, *Htra3*, *Kera*, *Ssc5d*, *Tnmd*, and *Zfp185* in the autopod and zeugopod regions of the E15.5 forelimbs in wildtype and $Scx^{-/-}$ littermates by section in situ hybridization analysis. Black arrow points to signal in non-tendon connective tissues in the dorsal region. Black arrowhead points to tendon tissues in the ventral region. Red arrowhead points to tendon tissues in the dorsal region. Yellow arrows point to signal in perichondral tissues.
